# Dietary 4-Hydroxy-2,5-Dimethyl-3(2H)-Furanone Supplementation in Hu Sheep: Implications on Fecal and Rumen Microbiota

**DOI:** 10.3390/ani16142212

**Published:** 2026-07-16

**Authors:** Chang Liu, Xinfeng Chen, Chuanpei Fu, Kehui Ouyang, Mingren Qu, Qinghua Qiu

**Affiliations:** Jiangxi Province Key Laboratory of Animal Nutrition and Feed, College of Animal Science and Technology, Jiangxi Agricultural University, Nanchang 330045, China

**Keywords:** gut microbiota, predicted functional pathway, microbial diversity, microbial ecology, ruminants, sheep

## Abstract

This study aimed to investigate the effects of dietary 4-hydroxy-2,5-dimethyl-3(2H)-furanone (HDMF) supplementation on ruminal and fecal microbial diversity, community composition, and predicted functional pathways. A total of 24 four-month-old female Hu sheep with similar body weights (20.6 ± 0.5 kg) were randomly allocated to two groups, with six replicates per group and two sheep per replicate. One group was fed a basal diet (CON), and the other received the same basal diet supplemented with 100 mg/kg HDMF. The results showed that ruminal microbial alpha diversity did not differ between the CON and HDMF groups (*p* > 0.05), whereas fecal microbial richness and evenness were higher in the CON group than in the HDMF group (*p* < 0.05). Beta diversity of ruminal microbiota did not differ between groups (R = 0.0667, *p* = 0.217), whereas fecal microbiota exhibited significant differences (R = 0.7333, *p* = 0.002). Dietary HDMF supplementation increased the relative abundances of Lachnospiraceae and *Oribacterium* in the rumen, elevated Spirochaetota and *Lachnospiraceae AC2044 group* in feces, and concurrently decreased Desulfobacterota and *Alistipes* in feces. Correspondingly, PICRUSt2-based functional prediction indicated putative associations between HDMF-induced microbial shifts and altered functional pathways, including increased relative abundances of amino acids biosynthesis and ABC transporter pathways, concomitant with decreased relative abundances of glycolysis/gluconeogenesis and pyruvate metabolism pathways in feces. These results suggest that dietary HDMF supplementation was associated with more pronounced alterations in fecal microbiota than in ruminal microbiota. This study provides a preliminary microbial ecology perspective on gastrointestinal microbiota in response to dietary HDMF supplementation in ruminants.

## Simple Summary

4-Hydroxy-2,5-dimethyl-3(2H)-furanone (HDMF) is a natural flavor compound contributing to the sweet, caramel-like aroma of ripe strawberries that is widely used in food products. Recent studies suggest that it may also benefit animals when added to ruminant feed. This study investigated how dietary HDMF supplementation affects gastrointestinal microbiota in Hu sheep. We found that HDMF had minimal impact on rumen microbiota but altered fecal microbiota, characterized by increased relative abundances of *Lachnospiraceae AC2044 group* and Spirochaetota, and decreased relative abundances of Desulfobacterota and *Alistipes*. Functionally, PICRUSt2 predicted increased relative abundances of amino acid biosynthesis and ABC transporter pathways, concomitant with decreased relative abundances of glycolysis/gluconeogenesis and pyruvate metabolism pathways. These results indicate that HDMF exerts more pronounced effects on fecal than on ruminal microbiota, providing a preliminary microbial ecology perspective on gastrointestinal microbiota in response to dietary HDMF supplementation in ruminants.

## 1. Introduction

Feed additive strategies constitute a fundamental pillar of contemporary livestock production systems, aimed at optimizing productive performance, metabolic efficiency, and physiological resilience [1]. While traditional additive classes, including antimicrobial growth promoters, exogenous enzymes, and organic acids, have historically dominated nutritional interventions, the escalating demand for antibiotic-free production models has intensified exploration into naturally occurring bioactive substances [2]. Within this expanding category, volatile flavor compounds derived from plant sources, long appreciated primarily for their sensory attributes, are progressively being repositioned as physiologically functional constituents capable of modulating oxidative status, inflammatory pathways, and microbial ecology [3]. This re-conceptualization has stimulated systematic evaluation of specific aroma molecules as dual-purpose feed supplements that may concurrently influence palatability and host biological processes.

4-Hydroxy-2,5-dimethyl-3(2H)-furanone (HDMF) emerges as a representative compound within this bioactive flavorant group. This heterocyclic ketone occurs endogenously in multiple fruit species, notably strawberry (*Fragaria* spp.) and pineapple (*Ananas comosus*), and is additionally generated through non-enzymatic browning reactions during thermal food processing [4]. Although predominantly investigated within flavor chemistry and food technology, HDMF has recently attracted attention for biological properties extending beyond sensory attributes. In addition to its substantial free radical scavenging capacity and suppression of lipid peroxidation, HDMF functions as a quorum sensing inhibitor that reduces biofilm formation and virulence factor expression, and may exert selective pressure on bacterial communities favoring beneficial species over pathogens [5,6,7]. Collectively, these findings suggest HDMF may interact with host tissues and microflora beyond olfactory functions.

The application of HDMF in animal production is currently in its infancy. To the best of our knowledge, peer-reviewed studies investigating HDMF as a feed additive in poultry, monogastric mammals, or aquatic animals are scarce, only with our preceding investigations in ruminants representing the primary available evidence [8,9]. The metabolic fate and pharmacokinetic profile of HDMF in animals have been partially characterized in rodents. Following oral administration to mice, HDMF is rapidly absorbed, appearing in plasma within 5 min and peaking between 15 and 45 min, with complete clearance within 2 h, indicating efficient biotransformation or elimination [10]. The hepatic metabolism of HDMF in animals, however, remains poorly characterized; available human data indicate that glucuronidation is the predominant pathway, forming the *β*-D-glucuronide conjugate as the major metabolite [11], but direct evidence in animal models is lacking. In vitro transport studies using Caco-2 cell monolayers demonstrate that intestinal absorption occurs via passive diffusion without saturable uptake [12]. The metabolic fate and pharmacokinetic behavior of HDMF in ruminants remain entirely uncharacterized.

Ruminants harbor two distinct microbial communities in the rumen and the hindgut. Though connected by digesta flow, these compartments differ markedly in their microbial composition and function [13]. The rumen chamber sustains an extraordinarily dense, phylogenetically diverse consortium of microorganisms executing anaerobic fermentation of ingested feedstuffs into volatile fatty acids (VFA), which constitute the principal energy substrate for the host animal [14]. Distal to this primary fermentation site, the large intestinal microbiome fulfills complementary functions encompassing immune system education, colonization resistance against enteric pathogens, and salvage fermentation of otherwise indigestible substrates [15]. These regional microbiota exhibit pronounced divergence in community composition, metabolic potential, and environmental physicochemistry, attributable to variations in oxidation–reduction status, available substrate profiles, digesta residence times, and epithelial secretory inputs [16,17]. This regional difference indicates that bioactive substances delivered by diet may have different effects depending on where they act in the digestive tract.

Phytochemical modulation of ruminant digestive microbiota has been substantiated across an array of molecular categories, including terpenoid essential oils, glycosidic saponins, polyphenolic tannins, and flavonoid derivatives [18]. Nevertheless, the capacity of such compounds to directly alter ruminal microbial ecology is frequently compromised by their inherent chemical instability under strictly anaerobic, strongly reducing conditions characteristic of the rumen environment, coupled with their vulnerability to enzymatic bioconversion by resident microorganisms [19,20]. Extensive structural alteration, microbial assimilation, or particulate binding during ruminal transit frequently diminishes the concentration of intact bioactive molecules reaching the caudal intestine [21,22]. Consequently, the hindgut may be exposed to distinctive compound profiles, comprising surviving parent molecules, microbial transformation products, or host-derived metabolites, potentially engendering differential ecological effects relative to the rumen [23,24].

Our preceding investigation revealed that dietary HDMF inclusion enhanced growth performance, nutrient digestibility, and serum antioxidant capacity in sheep [8,9]. Strikingly, this supplementation regime exerted compartment-specific effects on fermentation indices: ruminal ammonia nitrogen concentration was altered, whereas VFA concentration and proportions remained unchanged; conversely, fecal VFA concentration and proportions were perturbed, whereas ammonia nitrogen was unaffected [9]. This asymmetric response pattern, differential effects on nitrogen metabolism in the rumen versus carbohydrate fermentation in the hindgut, suggests that HDMF or its bioactive derivatives reach both compartments but elicit distinct functional consequences mediated by compartment-specific microbial assemblages. The microbial ecological mechanisms underlying these compartment-specific fermentation differences remain unknown and warrant further investigation.

Accordingly, the present investigation was undertaken to compare the effects of HDMF on ruminal and fecal bacterial diversity and community structure in Hu sheep. Our hypothesis posited that HDMF would exert differential effects on fecal versus ruminal microbiota, consistent with our previously observed fermentation differences. These findings are expected to elucidate the gastrointestinal distribution of HDMF’s microbiological effects and support its practical application as a functional feed additive in ruminant production.

## 2. Materials and Methods

### 2.1. Experimental Design

All procedures involving animals were conducted in accordance with ethical standards and approved by the Institutional Animal Care and Use Committee of Jiangxi Agricultural University (approval no. JXAULL-20240336). This experiment was conducted as part of a larger project investigating the effects of dietary supplementation with HDMF on sheep production and its underlying mechanisms. A subset of the experimental animals was shared with Qiu et al. [9]; however, the research objectives and analytical scope were distinct. Qiu et al. [9] primarily assessed growth performance, nutrient digestion, and gastrointestinal fermentation, whereas the present study focused exclusively on characterizing the gastrointestinal microbiota. A total of 24 healthy Hu ewe lambs (20.6 ± 0.5 kg BW; 122.7 ± 0.6 d of age) were randomly assigned to two groups using a completely randomized design based on initial body weight. Each group comprised 6 pens with 2 sheep per pen, with the pen serving as the experimental unit (6 replicates per group). The sample size (*n* = 6 per treatment) was determined by power analysis based on average daily gain data from our preceding four-dose–response study [9] (Cohen’s f = 0.80, alpha = 0.05, statistical power = 0.80). The control group was fed a basal diet (CON), whereas the treatment group was fed the same basal diet supplemented with HDMF at a dose of 100 mg/kg of feed on a dry matter basis (HDMF). The dietary HDMF supplementation dosage (100 mg/kg) was selected based on our preceding dose–response study in Hu sheep [9], wherein this level elicited optimal improvements in growth performance, nutrient digestibility, and serum antioxidant capacity. The source of HDMF and its incorporation into the diet followed the procedures outlined by Qiu et al. [9]. Ingredients and chemical composition of the basal diet are shown in Table 1. This 75 d study included a 15 d adaptation phase and a 60 d experimental period. Animals received feed twice daily at 08:00 and 18:00 h, with free access to water. Feed provision was regulated according to prior-day consumption to maintain orts at 5–10% of the amount offered.

### 2.2. Sample Collection

Following the procedure outlined by Paz et al. [25], rumen contents were sampled during the final two consecutive days of the trial using oral esophageal tubing. To avoid salivary contamination, the first 100 mL of the collected material was discarded. The subsequent sample was then passed through four layers of gauze to obtain rumen fluid. During the 5-day period preceding the end of the trial, fecal collection was performed every four hours from each animal, yielding 6 samples per day. These 30 time-point composites (6 samples/day × 5 days) were thoroughly homogenized and merged into one representative composite sample per animal. All fecal and rumen fluid samples were preserved at −80 °C pending DNA extraction.

### 2.3. DNA Extraction and Amplicon Sequencing

A total of 24 composite samples (12 ruminal fluid and 12 feces) were processed for genomic DNA extraction using the QIAGEN PowerFecal Pro DNA Kit (QIAGEN, Hilden, Germany). Each composite sample represented an equal-volume pool of ruminal fluid or an equal-mass pool of feces from two sheep within the same pen, resulting in six replicates per group for each sample type (*n* = 6). Extracted DNA quality and concentration were assessed by three complementary approaches: fluorometric quantification with a Qubit 3.0 instrument (Life Technologies, Carlsbad, CA, USA), spectrophotometric purity evaluation via NanoDrop ND-2000 (Thermo Fisher Scientific, Waltham, MA, USA), and integrity verification through 1% agarose gel electrophoresis. For bacterial community profiling, the near-full-length 16S rRNA gene was targeted with primer pair 27F/1492R (5′-AGRGTTYGATYMTGGCTCAG-3′ and 5′-RGYTACCTTGTTACGACTT-3′), with sample-indexing achieved through 16 bp barcoded primers. Each 25-μL amplification contained 5 μL 5× KAPA HiFi Buffer, 0.5 μL KAPA HiFi HotStart polymerase (1 U/μL), 0.5 μL 10 mM dNTPs, 1 μL 10 μM forward primer, 1 μL 10 μM reverse primer, 1.0 μL template DNA, and 16 μL nuclease-free water. Thermal cycling commenced with 5 min at 95 °C, proceeded through 30 three-step cycles (95 °C, 30 s; 57 °C, 30 s; 72 °C, 60 s), and concluded with 5 min at 72 °C. Amplicons were size-selected and purified with AMPure XP beads (Beckman Coulter, Brea, CA, USA), then quantified fluorometrically (Quant-iT dsDNA HS, Invitrogen, Carlsbad, CA, USA). SMRTbell libraries were constructed using the Express Template Prep Kit 2.0 (Pacific Biosciences, Menlo Park, CA, USA), quality-controlled and validated, and sequenced on a PacBio Sequel II system by BAXBio Technology Co., Ltd. (Beijing, China). Raw reads were archived in NCBI SRA under BioProject PRJNA1276234 for rumen samples and PRJNA1276245 for fecal samples.

### 2.4. Sequencing Data Analysis

The raw PacBio reads were demultiplexed and converted to circular consensus sequences (CCS/HiFi reads) via SMRT Link v13.1. Sequencing depth ranged from 33,013 to 42,232 raw reads per sample for ruminal fluid and from 32,308 to 41,597 raw reads per sample for feces, yielding 28,114 to 39,421 and 25,556 to 32,067 effective reads, respectively, after quality filtering, denoising, and chimera removal. Rarefaction curves confirmed that all samples approached saturation by 20,000 sequences (Figure S1). Samples were rarefied to 28,114 reads per sample for ruminal fluid and 25,556 reads per sample for feces for downstream analysis. Amplicon sequence variants (ASVs) were inferred using the DADA2 pipeline (v1.30.0), followed by taxonomic assignment against the SILVA 138 reference database with the classify-sklearn classifier implemented in QIIME 2 (v2023.9). Within-sample diversity was characterized by multiple alpha-diversity indices: Chao1, ACE, Shannon index, Simpson index, Faith’s phylogenetic diversity (PD), and Pielou’s evenness, all computed through the QIIME 2 “diversity alpha” plugin. Beta diversity was visualized by non-metric multidimensional scaling (NMDS) based on Bray–Curtis dissimilarities, implemented through the vegan package (v2.6-4) in R. Between-group compositional similarity was formally tested by analysis of similarities (ANOSIM), also executed via vegan. Differentially abundant taxa across taxonomic ranks were identified by linear discriminant analysis effect size (LEfSe), which first applied the Kruskal–Wallis rank-sum test to screen for features with significant abundance heterogeneity, then estimated each taxon’s discriminatory contribution through linear discriminant analysis (LDA); taxa achieving an LDA score > 3.0 were considered robust biomarkers. Functional potential of the microbial communities was inferred using phylogenetic investigation of communities by reconstruction of unobserved states 2 (PICRUSt2, v2.5.2) against the kyoto encyclopedia of genes and genomes (KEGG) database, enabling prediction of functional pathway alterations in response to HDMF supplementation in both ruminal and fecal microbiota.

### 2.5. Statistical Analysis

Microbiome analysis was conducted on pen-level composite samples (*n* = 6 per treatment for each sample type). The normality of alpha-diversity indices was assessed using the Shapiro–Wilk test. For normally distributed indices (Chao1, ACE, Shannon index, Faith’s PD, and Pielou’s evenness), between-group differences were evaluated using Welch’s independent two-sample *t*-test. The Simpson index, which did not meet the normality assumption, was analyzed using the Wilcoxon rank-sum test (Mann–Whitney U test). All *p*-values were adjusted for multiple comparisons using the Benjamini–Hochberg false discovery rate (FDR) method (adjusted *p* < 0.05 considered significant). Differential abundance analysis was performed using ALDEx2 (v1.34.0) in R (v4.3.1; R Core Team, Vienna, Austria, 2023), accounting for the compositional nature of microbiome data. Briefly, 128 Monte-Carlo instances were drawn from the Dirichlet distribution, and between-group differences were evaluated using Welch’s *t*-test for each instance. Statistical significance was assessed using Benjamini–Hochberg with adjusted *p*-values, and adjusted *p* < 0.05 was considered significant.

## 3. Results

### 3.1. Microbial Alpha-Diversity

The effects of dietary HDMF supplementation on the alpha diversity of ruminal and fecal microbiota are presented in Table 2 and Table 3, respectively. HDMF had no effect on the richness or diversity of rumen microbiota (*p* > 0.05); however, it reduced the Chao1, ACE, Shannon index, Simpson index, and Pielou’s evenness in fecal microbiota (*p* < 0.05).

### 3.2. Microbial Community Composition

The effects of dietary HDMF supplementation on the relative abundances of ruminal and fecal microbiota at the phylum level are presented in Table 4 and Table 5, respectively. HDMF had no significant effect on any phylum with a relative abundance above 0.1% in ruminal microorganisms (*p* > 0.05). In fecal microbiota, however, HDMF supplementation increased the relative abundance of Spirochaetota while decreasing that of Desulfobacterota.

The effects of dietary HDMF on the relative abundances of ruminal and fecal microbiota at the genus level are shown in Table 6 and Table 7, respectively. HDMF increased the relative abundances of *unclassified_Lachnospiraceae* and *Oribacterium* in the rumen (*p* < 0.05). Similarly, in fecal microbiota, HDMF increased the relative abundances of *unclassified_Lachnospiraceae*, *Treponema*, and *Lachnospiraceae_AC2044_group*, while reducing the relative abundances of *Alistipes* and *NK4A214_group* (*p* < 0.05).

### 3.3. Microbial Beta-Diversity

**Figure 1:**
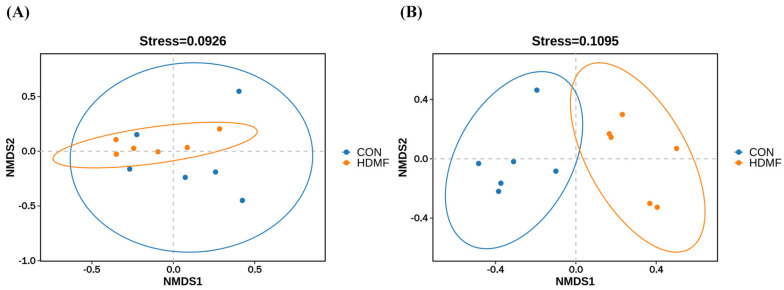
Non-metric multidimensional scaling analysis of ruminal microbiota (**A**) and fecal microbiota (**B**) between the group that received only the basal diet (CON) and the group that received the basal diet supplemented with 100 mg/kg of 4-hydroxy-2,5-dimethyl-3(2H)-furanone (HDMF).

The NMDS plot of ruminal microbiota (Figure 1A) showed substantial overlap between the CON and HDMF groups, and ANOSIM confirmed that the between-group difference was not significant (R = 0.0667, *p* = 0.217). In contrast, the NMDS plot of fecal microbiota (Figure 1B) revealed a discernible separation between the two groups, with ANOSIM indicating a significant difference (R = 0.7333, *p* = 0.002).

### 3.4. Marked Microbiota

**Figure 2:**
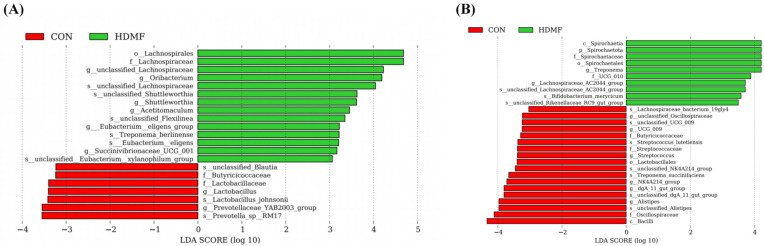
Linear discriminant analysis effect size bar chart of ruminal microbiota (**A**) and fecal microbiota (**B**) between the group that received only the basal diet (CON) and the group that received the basal diet supplemented with 100 mg/kg of 4-hydroxy-2,5-dimethyl-3(2H)-furanone (HDMF).

LEfSe analysis of rumen microbiota identified 21 differential biomarkers (Figure 2A). Of these, 14 were enriched in the HDMF group: o__Lachnospirales, f__Lachnospiraceae, g__*unclassified_Lachnospiraceae*, g__*Oribacterium*, s__*unclassified_Lachnospiraceae*, s__*unclassified_Shuttleworthia*, g__*Shuttleworthia*, g__*Acetitomaculum*, s__*unclassified_Flexilinea*, g__*Eubacterium_eligens_group*, s__*Treponema_berlinense*, s__*Eubacterium_eligens*, g__*Succinivibrionaceae_UCG_001*, and s__*unclassified_Eubacterium_xylanophilum_group*. The remaining 7 biomarkers were enriched in the CON group: s__*unclassified_Blautia*, f__Butyricicoccaceae, f__Lactobacillaceae, g__*Lactobacillus*, s__*Lactobacillus_johnsonii*, g__*Prevotellaceae_YAB2003_group*, and s__*Prevotella_*sp_RM17.

LEfSe analysis of fecal microbiota identified 28 differential biomarkers (Figure 2B). Of these, 10 were enriched in the HDMF group: c__Spirochaetia, p__Spirochaetota, f__Spirochaetaceae, o__Spirochaetales, g__*Treponema*, f__UCG_010, g__*Lachnospiraceae_AC2044_group*, s__*unclassified_Lachnospiraceae_AC2044_group*, s__*Bifidobacterium_merycicum*, and s__*unclassified_Rikenellaceae_RC9_gut_group*. The remaining 18 were enriched in the CON group: s__*Lachnospiraceae_bacterium_19gly4*, g__*unclassified_Oscillospiraceae*, s__*unclassified_UCG_009*, g__*UCG_00*9, f__Butyricicoccaceae, s__*Streptococcus_lutetiensis*, f__Streptococcaceae, g__*Streptococcus*, o__Lactobacillales, s__*unclassified_NK4A214_group*, s__*Treponema_succinifaciens*, g__*NK4A214_group*, g__*dgA_11_gut_group*, s__*unclassified_dgA_11_gut_group*, g__*Alistipes*, s__*unclassified_Alistipes*, f__Oscillospiraceae, and c__Bacilli.

### 3.5. Predicted Metabolic Function

The effects of dietary HDMF supplementation on the relative abundances of predicted functional pathways (inferred using PICRUSt2) in ruminal and fecal microbiota are presented in Table 8 and Table 9, respectively. HDMF had no significant effect on any predicted functional pathway with a relative abundance above 1% in rumen microorganisms (*p* > 0.05). In fecal microbiota, however, HDMF supplementation increased the relative abundances of biosynthesis of amino acids and ABC transporters, while decreasing the relative abundances of microbial metabolism in diverse environments, carbon metabolism, glycolysis/gluconeogenesis, and pyruvate metabolism (*p* < 0.05).

## 4. Discussion

The present study provides the first in vivo evidence that dietary HDMF supplementation exerts differential effects on ruminal and fecal microbial communities in sheep, characterized by a significant reduction in fecal bacterial diversity yet remarkable stability in ruminal alpha diversity. Alpha diversity, which encompasses species richness and evenness, serves as a fundamental indicator of ecological stability and functional capacity within ruminant microbial ecosystems [26]. The ruminal microbiota demonstrated considerable ecological stability, potentially consistent with extensive functional redundancy among constituent populations, rapid dilution of ingested bioactive compounds, and efficient microbial enzymatic conversion of plant-derived furanones [27,28,29]. Conversely, the fecal microbial community exhibited pronounced susceptibility, which may be associated with progressive enrichment of the compound along the gastrointestinal tract, extended residence time within the hindgut, and interference with quorum sensing-mediated bacterial communication [30,31,32]. Together with our previous finding of a concomitant decrease in fecal ammonia nitrogen concentration [9], the reduced fecal alpha diversity observed in the present study is compatible with the hypothesis that HDMF may alter proliferation and epithelial adhesion of specific sensitive bacterial taxa within the hindgut. Such a pattern could reflect diminished interspecific competition, permitting HDMF-tolerant populations to proliferate while outcompeting less resistant species, and potentially contributing to the observed decline in overall community diversity. It is proposed that the divergent diversity patterns between these compartments are not necessarily attributable to intrinsic differences in microbial tolerance per se, but may be influenced by variable HDMF bioavailability governed by regional physicochemical conditions and digesta transit dynamics.

Despite these compartment-specific diversity responses, taxonomic analysis revealed that HDMF induced changes in select bacterial genera across both ruminal and fecal communities. The caramel and fruity aroma of HDMF stimulated a numerical increase in feed intake as seen in our previous study [9]. Given that dietary fiber content was identical between the CON and HDMF groups, the latter consumed a greater total amount of fiber [9]. Members of the phylum Spirochaetota exhibit cellulase and hemicellulase activities, enabling sustained fiber fermentation throughout the post-ruminal gastrointestinal tract [33]. This putative mechanism is indirectly supported by the numerically elevated acetate concentrations observed in both ruminal and fecal samples [9]. The concomitant decrease in Desulfobacterota abundance in the HDMF group is hypothetically consistent with a putative shift in electron flow from dissimilatory sulfate reduction toward fermentative pathways dominated by Spirochaetota [34]. This interpretation remains speculative, as electron flow dynamics were not directly measured in this study. This ecological transition is hypothesized to potentially reflect enhanced fiber fermentation, which may create conditions conducive to hindgut acidification and altered competitive dynamics for electron donors (H_2_ and lactate), as indirectly suggested by numerically elevated acetate concentrations reported in our previous work [9]. Following the disruption of cellulose crystallinity and release of soluble oligosaccharides by Spirochaetota, Lachnospiraceae members utilize these partially hydrolyzed substrates to further degrade amorphous cellulose and pectin, yielding butyrate [35]. As the principal energy source for intestinal epithelial cells, butyrate is reported to strengthen mucosal barrier integrity [36]. In the present study, the relative abundance of Lachnospiraceae increased in both the rumen and feces, concomitant with an increase in Spirochaetota and a decrease in Desulfobacterota. These concurrent changes are consistent with a coordinated ecological shift toward fiber-fermenting communities in the HDMF group [9]. The increased relative abundance of *Oribacterium*, an amino acid-fermenting genus within the Firmicutes, in the rumen of the HDMF group suggests enhanced protein turnover and ammonia generation, as evidenced by the elevated microbial crude protein and ammonia nitrogen concentrations in our previous work [9]. Notably, *Oribacterium* has been reported to possess adhesive properties potentially conducive to biofilm formation [37], and its increased abundance in the present study coincided with elevated ruminal biofilm biomass observed in our previous work [9]. This metabolic activity likely complemented the concurrent proliferation of fiber-degrading taxa (Spirochaetota and Lachnospiraceae) by supplying ammonia nitrogen for microbial protein synthesis, while the enhanced biofilm matrix may have facilitated spatial proximity and metabolite exchange among these fermentative communities. Similarly, as an amino acid-fermenting bacterium, *Alistipes* exhibited a decreased relative abundance in feces. Given its extreme oxygen sensitivity and narrow pH optimum, *Alistipes* may be outcompeted by acid-tolerant, fiber-degrading taxa such as Spirochaetota and Lachnospiraceae under high substrate load conditions [38]. However, the ecological role of *Alistipes* in ruminant hindgut fermentation remains poorly characterized, warranting further isolation and functional studies.

LDA revealed a more comprehensive spectrum of differentially abundant microbiota in rumen and feces. In the rumen, the HDMF group was enriched in *Acetitomaculum* and *Eubacterium group*, both of which are associated with fiber fermentation and acetate production [39,40]. Concurrently, *Flexilinea* and *Shuttleworthia*, taxa reported to carry adhesive properties potentially conducive to biofilm formation [41,42], were also elevated, consistent with the enhanced ruminal biofilm content observed in the HDMF group reported in our previous work [9]. Given the numerically higher feed intake in the HDMF group as reported in our previous work [9], this potentially suggests that greater feed intake may be associated with the assembly of biofilm-embedded degradative communities, where spatial proximity could facilitate metabolite exchange among fermenters. The functional relevance of this putative restructuring is further supported by the higher digestibility of dry matter, crude protein, and ether extract of the HDMF group in our previous findings [9], indicating that the proliferation of these fiber-fermenting, biofilm-forming taxa was associated with improved nutrient utilization. In the feces, the HDMF group was enriched in *Bifidobacterium merycicum* and *Rikenellaceae RC9 gut group*, taxa characteristic of the hindgut fermentation compartment. *Bifidobacterium merycicum* specializes in oligosaccharide utilization, whereas the *Rikenellaceae RC9 gut group* is adept at peptide and amino acid fermentation [43,44]. Their proliferation likely reflects the increased flux of partially digested fiber and protein into the hindgut under elevated feed intake, as shown in Qiu et al. [9], in which they function as secondary fermenters potentially complementing the fiber-degrading activity of Spirochaetota and Lachnospiraceae. It is hypothesized that the enrichment of *Rikenellaceae RC9 gut group* alongside the decline in *Alistipes* may reflect a community restructuring within the amino acid-fermenting guild, as acid-tolerant taxa may have prevailed under intensified fermentation conditions. It is acknowledged that the ecological role of *Alistipes* is context-dependent, with certain species exhibiting beneficial functions such as butyrate production and anti-inflammatory effects in specific hosts, while others may contribute to protein fermentation under suboptimal conditions [45]. In the present study, the decline in *Alistipes* was observed alongside enhanced fermentation intensity in the HDMF group as reported in Qiu et al. [9]. Whether this shift reflects adaptation to altered fermentation kinetics or represents ecological replacement remains to be determined. The CON group was characterized by a cross-compartment enrichment in taxa indicative of divergent fermentation modes. In the rumen, the over-representation of Lactobacillaceae and *Prevotella* sp. RM17 pointed to lactic acid accumulation risk and excessive protein degradation, respectively [46,47]. In feces, the dominance of Streptococcaceae reflected lactic acid fermentation, while the enrichment of *Treponema succinifaciens* indicated succinate-type fermentation [48]. It is recognized that *Treponema* encompasses species with diverse ecological roles, including fiber degradation and cross-feeding interactions [49]; however, in the context of the present hindgut environment, the succinate-oriented metabolism represented by *Treponema succinifaciens* is energetically less efficient for the host compared to the acetate- and butyrate-oriented fermentation observed in the HDMF group [9,50,51]. Notably, both compartments exhibited increased relative abundances of taxa with poorly characterized metabolic functions, including *Oscillospiraceae UCG-009*, *NK4A214 group*, and *dgA-11 gut group*. The prevalence of lactic acid bacteria across the gastrointestinal tract was observed in the CON group, whereas the HDMF group showed higher relative abundances of taxa previously associated with the acetate and butyrate production in fiber-degrading communities. These compositional differences are not sufficient to infer functional consequences without metabolomic or metatranscriptomic validation.

The aforementioned differential microorganisms were associated with predicted functional pathways. However, it is critical to emphasize that all functional interpretations presented below are derived from PICRUSt2 predictions rather than direct metagenomic or metabolomic measurements. These pathways should be regarded as putative hypotheses requiring experimental validation. In feces, the HDMF group exhibited elevated amino acid biosynthesis and ABC transporter pathways alongside reduced carbon metabolism, glycolysis/gluconeogenesis, and pyruvate metabolism. The upregulation of amino acid biosynthesis is inferred to reflect enhanced nitrogen assimilation by *Rikenellaceae RC9 gut group*, a function commonly associated with Rikenellaceae members, while the elevated ABC transporter signature is hypothesized to align with oligosaccharide utilization characteristic of the genus *Bifidobacterium* [40,41]. The concomitant decline in glycolysis and pyruvate metabolism is consistent with suppression of lactic acid fermentation, paralleling the dominance of lactic acid bacteria in the CON group. Furthermore, the reduction in microbial metabolism in diverse environments is speculated to suggest ecosystem maturation, wherein metabolic specialization may replace functional redundancy. This hypothetical transition, characterized by channeling carbon toward acetate and butyrate rather than lactate, provides a speculative mechanistic framework for the improved nutrient digestibility in the HDMF group as shown in previous work [9].

This study provides an integrated multi-compartment analysis of ruminal and fecal microbiota. The results suggest that HDMF is associated with relatively greater impact on hindgut compared with ruminal microbial communities, although the ecological and functional consequences of these compositional shifts remain to be fully elucidated. The combination of 16S rRNA gene sequencing with PICRUSt2 functional pathway prediction was employed to explore putative functional differences beyond taxonomy; these computational inferences are compatible with potential associations between changes in microbial community structure and altered nitrogen assimilation and carbohydrate metabolism pathways. It is important to emphasize that PICRUSt2 predictions are based on inferred genomic content and existing reference databases with limited accuracy for rumen ecosystems, particularly due to incomplete genomic characterization of many taxa and strain-level metabolic variations not captured by 16S-based inference. Consequently, the observed pathway shifts should be regarded as putative hypothesis rather than definitive biological outcomes; the inferred link between taxonomic and functional changes remains strictly correlative, and any mechanistic interpretations should be treated as testable hypothesis requiring experimental validation through metagenomic, metatranscriptomic, or metabolomic approaches. Moreover, the functional relationships between the host and gastrointestinal bacteria could not be established. The phenotypic data from this cohort were previously published in a subscription journal [9], and the permissibility of secondary correlation-based re-analysis under the original publication agreement remains uncertain. Future prospective studies employing Spearman correlation and Mantel tests with simultaneously collected microbiota and host metabolic data will be needed to robustly characterize these associations. Furthermore, this study was conducted using female Hu sheep, a breed adapted to high-forage diets; therefore, the observed associations should be interpreted with caution and may not be generalizable to other ruminant species or production systems, particularly feedlot finishing systems fed high-concentrate diets.

## 5. Conclusions

In summary, dietary HDMF supplementation reduced fecal microbial alpha diversity without affecting ruminal microbial alpha diversity. HDMF increased the relative abundances of Lachnospiraceae and *Oribacterium* in the rumen, elevated Spirochaetota and *Lachnospiraceae AC2044 group* in feces, and concurrently decreased Desulfobacterota and *Alistipes* in feces. PICRUSt2-based functional prediction indicated that the fecal microbiota of the HDMF group was putatively associated with increased relative abundances of amino acids biosynthesis and ABC transporter pathways, alongside decreased relative abundances of glycolysis/gluconeogenesis and pyruvate metabolism pathways in feces. These findings suggest compartment-specific responses of ruminal and fecal microbiota to dietary HDMF supplementation, potentially offering a preliminary microbial ecology perspective on gastrointestinal microbiota in response to dietary HDMF supplementation in ruminants.

## Figures and Tables

**Table 1:** Feed ingredients and their chemical composition in the basal diet.

Ingredient	Proportion, g/kg	Nutritional Components	Value
Corn	281.5	Metabolizable energy, Mcal/kg	2.52
Soybean meal	80.0	Crude protein, %	13.26
Wheat bran	160.0	Neutral detergent fiber, %	35.97
Wheat straw	150.0	Acid detergent fiber, %	22.34
Peanut straw	300.0	Ether extract, %	2.85
Calcium hydrogen phosphate	8.5	Calcium, %	0.89
Limestone	5.0	Phosphorus, %	0.51
Salt	5.0		
Premix ^1^	10.0		

^1^ Premix provided the following per kg of DM: 1400 mg of Fe, 1200 mg of Zn, 250 mg of Cu, 900 mg of Mn, 100,000 IU of vitamin A, 27,000 IU of vitamin D3, and 800 IU of vitamin E.

**Table 2:** Effect of dietary HDMF supplementation on the alpha diversity of rumen microbiota in Hu sheep.

Item	CON	HDMF	SEM	*p*-Value
Chao1	1285.99	1371.83	104.80	0.575
ACE	1291.90	1377.71	104.32	0.574
Shannon index	5.61	5.62	0.124	0.967
Simpson index	0.9858	0.9896	0.002	1.000
Faith’s phylogenetic diversity	64.57	68.67	3.286	0.400
Pielou’s evenness	0.7858	0.7802	0.017	0.831

Note: Data are presented as mean and standard error of the mean (SEM). CON, control; HDMF, dietary 4-hydroxy-2,5-dimethyl-3(2H)-furanone supplementation with 100 mg/kg.

**Table 3:** Effect of dietary HDMF supplementation on the alpha diversity of fecal microbiota in Hu sheep.

Item	CON	HDMF	SEM	*p*-Value
Chao1	2014.12	1816.70	54.344	0.030
ACE	2035.06	1833.59	54.342	0.028
Shannon index	6.63	6.17	0.075	0.005
Simpson index	0.9961	0.9918	0.001	0.004
Faith’s phylogenetic diversity	100.30	97.77	2.675	0.535
Pielou’s evenness	0.8720	0.8226	0.009	0.007

Note: Data are presented as mean and standard error of the mean (SEM). CON, control; HDMF, dietary 4-hydroxy-2,5-dimethyl-3(2H)-furanone supplementation with 100 mg/kg.

**Table 4:** Effect of dietary HDMF supplementation on the rumen bacterial community at the phylum level in Hu sheep.

Item	CON	HDMF	SEM	*p*-Value
Firmicutes	53.65	51.29	2.601	0.548
Bacteroidota	39.08	40.10	3.129	0.828
Actinobacteriota	4.93	5.83	2.601	0.240
Proteobacteria	0.69	0.68	0.181	0.987
Cyanobacteria	0.53	0.77	0.536	0.589
Desulfobacterota	0.23	0.49	0.110	0.128
Spirochaetota	0.18	0.35	0.058	0.058
Fibrobacterota	0.10	0.24	0.064	0.178
Verrucomicrobiota	0.21	0.06	0.080	0.485
Patescibacteria	0.13	0.02	0.030	0.065
Nanoarchaeota	0.08	0.01	0.038	0.589
Planctomycetota	0.03	0.05	0.018	0.818
Synergistota	0.05	0.01	0.015	0.937
Chloroflexi	0.02	0.02	0.014	0.310
Fusobacteriota	0.03	0.00	0.013	0.699

Note: Data are presented as mean and standard error of the mean (SEM). CON, control; HDMF, dietary 4-hydroxy-2,5-dimethyl-3(2H)-furanone supplementation with 100 mg/kg.

**Table 5:** Effect of dietary HDMF supplementation on the fecal bacterial community at the phylum level in Hu sheep.

Item	CON	HDMF	SEM	*p*-Value
Firmicutes	60.84	54.68	2.606	0.184
Bacteroidota	27.80	25.66	1.360	0.334
Spirochaetota	4.43	7.53	0.880	0.036
Actinobacteriota	2.26	6.78	2.478	1.000
Verrucomicrobiota	2.31	2.03	1.083	0.240
Fibrobacterota	0.49	2.18	0.521	0.065
Planctomycetota	1.18	0.69	0.279	0.248
Proteobacteria	0.29	0.21	0.085	0.558
Cyanobacteria	0.22	0.09	0.050	0.110
Elusimicrobiota	0.04	0.09	0.028	0.394
Campylobacterota	0.05	0.04	0.016	0.469
Patescibacteria	0.04	0.01	0.013	0.180
Desulfobacterota	0.04	0.01	0.008	0.009
Myxococcota	0.01	0.00	0.003	0.180

Note: Data are presented as mean and standard error of the mean (SEM). CON, control; HDMF, dietary 4-hydroxy-2,5-dimethyl-3(2H)-furanone supplementation with 100 mg/kg.

**Table 6:** Effect of dietary HDMF supplementation on the rumen bacterial community at the genus level in Hu sheep.

Item	CON	HDMF	SEM	*p*-Value
*Prevotella*	26.11	32.69	2.948	0.165
*Ruminococcus*	14.88	3.04	2.681	0.093
*Selenomonas*	3.33	5.27	1.638	0.423
*Sharpea*	2.87	5.36	2.017	0.132
*Unclassified_Selenomonadaceae*	3.09	4.51	1.740	0.180
*Olsenella*	1.94	4.09	1.600	0.132
*Unclassified_Lachnospiraceae*	1.24	4.34	0.872	0.015
*Prevotellaceae_UCG_001*	3.32	1.93	1.144	0.937
*Lachnospiraceae_NK3A20_group*	2.34	2.21	0.509	0.874
*Oribacterium*	0.43	3.76	0.572	0.002
*UCG_004*	3.38	0.23	1.003	0.180
*Succiniclasticum*	1.21	2.32	0.387	0.073
*Roseburia*	1.15	2.16	0.890	0.310
*Rikenellaceae_RC9_gut_group*	2.01	1.14	0.430	0.210
*Unclassified_Prevotellaceae*	1.98	0.76	0.678	0.589
*Bifidobacterium*	1.42	1.12	0.811	0.180
*Syntrophococcus*	1.10	1.10	0.528	0.310
*[Ruminococcus]_gauvreauii_group*	1.08	1.10	0.443	0.180

Note: Data are presented as mean and standard error of the mean (SEM). CON, control; HDMF, dietary 4-hydroxy-2,5-dimethyl-3(2H)-furanone supplementation with 100 mg/kg.

**Table 7:** Effect of dietary HDMF supplementation on the fecal bacterial community at the genus level in Hu sheep.

Item	CON	HDMF	SEM	*p*-Value
*Christensenellaceae_R_7_group*	14.55	13.35	1.540	0.485
*Bacteroides*	9.89	6.66	1.276	0.118
*Rikenellaceae_RC9_gut_group*	6.42	7.87	1.225	0.818
*UCG_005*	7.44	6.45	0.361	0.090
*Treponema*	4.42	7.51	0.877	0.036
*Bifidobacterium*	1.31	6.43	2.421	0.394
*Alistipes*	3.95	1.93	0.256	<0.001
*Unclassified_Lachnospiraceae*	2.23	3.40	0.284	0.033
*Ruminococcus*	2.20	3.01	0.399	0.589
*Unclassified_UCG_010*	1.90	3.18	0.646	0.240
*Unclassified_Muribaculaceae*	1.66	2.72	0.660	0.322
*Akkermansia*	2.11	1.89	1.096	0.310
*Lachnospiraceae_AC2044_group*	1.35	2.34	0.222	0.011
*Unclassified_[Eubacterium]_coprostanoligenes_group*	1.97	1.59	0.628	0.818
*UCG_002*	1.74	1.78	0.159	0.842
*Monoglobus*	1.47	1.45	0.311	0.977
*NK4A214_group*	1.83	0.89	0.236	0.019
*Fibrobacter*	0.49	2.18	0.521	0.065
*[Eubacterium]_siraeum_group*	1.21	1.19	0.277	0.956
*Unclassified_Ruminococcaceae*	0.97	1.22	0.099	0.178

Note: Data are presented as mean and standard error of the mean (SEM). CON, control; HDMF, dietary 4-hydroxy-2,5-dimethyl-3(2H)-furanone supplementation with 100 mg/kg.

**Table 8:** Effect of dietary HDMF supplementation on the relative abundances of the predicted functional pathways (inferred using PICRUSt2) in the rumen bacterial community of Hu sheep.

Item	CON	HDMF	SEM	*p*-Value
Metabolic pathways	17.31	17.25	0.110	0.724
Biosynthesis of secondary metabolites	8.06	8.07	0.018	0.723
Biosynthesis of antibiotics	5.90	5.85	0.032	0.324
Biosynthesis of amino acids	4.32	4.32	0.052	0.931
Microbial metabolism in diverse environments	3.72	3.71	0.016	0.490
Carbon metabolism	2.57	2.56	0.011	0.422
Ribosome	2.57	2.53	0.029	0.317
ABC transporters	2.29	2.40	0.083	0.394
Purine metabolism	2.13	2.13	0.015	0.980
Pyrimidine metabolism	1.85	1.83	0.017	0.469
Two-component system	1.73	1.76	0.032	0.394
Amino sugar and nucleotide sugar metabolism	1.26	1.25	0.018	0.726
Quorum sensing	1.17	1.21	0.029	0.405
Aminoacyl-tRNA biosynthesis	1.12	1.10	0.015	0.414
Glycolysis/Gluconeogenesis	1.12	1.09	0.017	0.217
Cysteine and methionine metabolism	1.04	1.01	0.010	0.160

Note: Data are presented as mean and standard error of the mean (SEM). CON, control; HDMF, dietary 4-hydroxy-2,5-dimethyl-3(2H)-furanone supplementation with 100 mg/kg.

**Table 9:** Effect of dietary HDMF supplementation on the relative abundances of the predicted functional pathways in the fecal bacterial community of Hu sheep.

Item	CON	HDMF	SEM	*p*-Value
Metabolic pathways	16.85	16.81	0.024	0.297
Biosynthesis of secondary metabolites	7.88	7.92	0.017	0.171
Biosynthesis of antibiotics	5.83	5.87	0.022	0.394
Biosynthesis of amino acids	4.20	4.31	0.027	0.016
Microbial metabolism in diverse environments	3.85	3.76	0.009	<0.001
ABC transporters	2.60	2.71	0.026	0.027
Carbon metabolism	2.64	2.56	0.011	<0.001
Ribosome	2.59	2.57	0.018	0.467
Purine metabolism	2.11	2.15	0.024	0.394
Two-component system	1.98	1.98	0.050	0.949
Pyrimidine metabolism	1.82	1.82	0.010	0.838
Quorum sensing	1.30	1.31	0.009	0.364
Aminoacyl-tRNA biosynthesis	1.14	1.17	0.015	0.485
Amino sugar and nucleotide sugar metabolism	1.13	1.13	0.007	0.825
Glycolysis/Gluconeogenesis	1.07	1.03	0.011	0.026
Pyruvate metabolism	1.07	1.01	0.022	0.041
Cysteine and methionine metabolism	1.01	1.03	0.006	0.136

Note: Data are presented as mean and standard error of the mean (SEM). CON, control; HDMF, dietary 4-hydroxy-2,5-dimethyl-3(2H)-furanone supplementation with 100 mg/kg.

## Data Availability

Raw reads were archived in NCBI SRA under BioProject PRJNA1276234 for rumen samples and PRJNA1276245 for fecal samples.

## Supplementary Materials

The following supporting information can be downloaded at https://www.mdpi.com/article/10.3390/ani16142212/s1, Figure S1: Rarefaction curves of ruminal (A) and fecal (B) microbiota.

## Abbreviations

The following abbreviations are used in this manuscript:

HDMF	4-Hydroxy-2,5-dimethyl-3(2H)-furanone
VFA	Volatile fatty acids
CCS	Circular consensus sequences
ASVs	Amplicon sequence variants
NMDS	Non-metric multidimensional scaling
ANOSIM	analysis of similarities
LEfSe	Linear discriminant analysis effect size
LDA	Linear discriminant analysis
PICRUSt2	Phylogenetic investigation of communities by reconstruction of unobserved states 2
KEGG	Kyoto encyclopedia of genes and genomes
