# Supplementary material for: Dietary 4-Hydroxy-2,5-Dimethyl-3(2H)-Furanone Supplementation in Hu Sheep: Implications on Fecal and Rumen Microbiota
Source: Animals (Basel). 2026 Jul 16;16(14):2212. doi: 10.3390/ani16142212 (PMC13406052; doi:10.3390/ani16142212)
Supplement: Supplementary file 1 [file animals-16-02212-s001.zip › animals-4369342-supplementary.pdf]

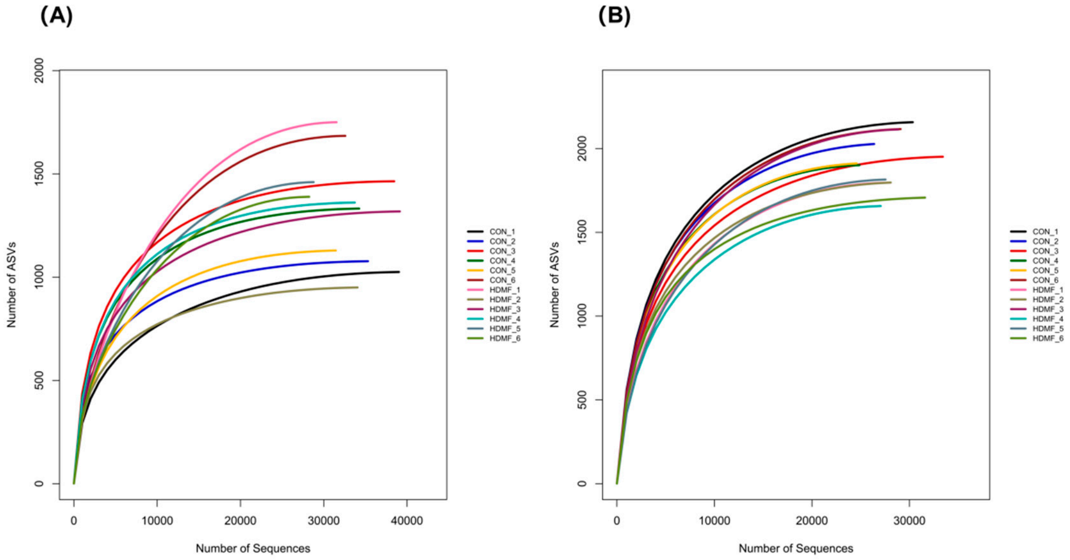

Figure S1. Rarefaction curves of ruminal (A) and fecal (B) microbiota. The x-axis indicates the number of sequences randomly subsampled per sample, and the y-axis indicates the observed number of ASVs. Each line represents an individual sample.
